# Supplementary material for: Lesion identification and malignancy prediction from clinical dermatological images
Source: Sci Rep. 2022 Sep 23;12:15836. doi: 10.1038/s41598-022-20168-w (PMC9508136; doi:10.1038/s41598-022-20168-w)
Supplement: Supplementary file 1 — Supplementary Information. [file 41598_2022_20168_MOESM1_ESM.pdf]

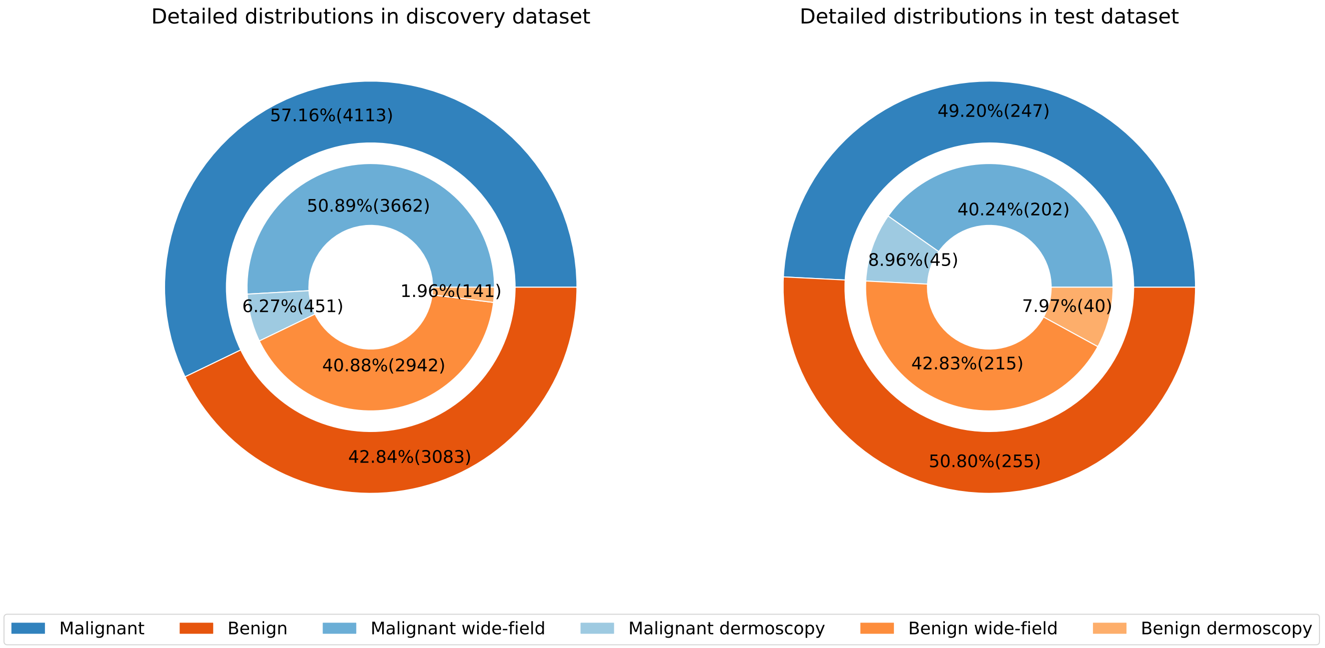

Supplementary Figure 1. The counts, ratios of malign and benign lesions in discovery and independent test set. Also, the counts, ratios of wild-field, dermoscopy lesions in malign or benign lesions.

		MEL	NV	BCC	AKIEC	BKL	DF	VASC	OB
$mAP_{@0.5}$	One-class	**0.888**	**0.837**	**0.777**	**0.852**	**0.829**	0.766	**0.802**	0.448
	Malignancy	0.856	0.799	0.693	0.825	0.705	**0.803**	0.802	**0.451**
	Sub-type	0.750	0.794	0.670	0.821	0.761	0.716	0.686	0.438
$mAP_{@0.75}$	One-class	0.374	**0.326**	0.276	**0.364**	**0.327**	0.229	0.244	**0.119**
	Malignancy	0.312	0.220	**0.284**	0.211	0.294	**0.318**	**0.525**	0.065
	Sub-type	**0.461**	0.271	0.201	0.264	0.289	0.256	0.406	0.083
$mAP_{@0.5,0.95}$	One-class	**0.449**	**0.398**	**0.368**	**0.412**	**0.407**	0.376	0.383	**0.188**
	Malignancy	0.391	0.338	0.319	0.369	0.353	**0.390**	**0.432**	0.176
	Sub-type	0.408	0.361	0.310	0.351	0.367	0.336	0.379	0.188
Recall	One-class	0.98	0.978	0.974	**0.975**	0.916	**1**	**0.8**	0.647
	Malignancy	**1**	**0.986**	**0.987**	0.967	**0.928**	0.909	0.8	0.588
	Sub-type	0.98	0.964	0.961	0.967	0.928	0.909	0.8	**0.706**

Supplementary Table 1. Sub-type lesion detection from smartphone (wide-field) and dermoscopy images. Performance is evaluated as the mean Average Precision (mAP) at three different thresholds: 0.5, 0.75 and $[0.5, 0.95]$, recall (sensitivity).

	IoU		
	One-class	Malignancy	Sub-type
MEL	$0.731_{(0.62,0.83)}$	$0.541_{(0.42,0.60)}$	$\mathbf{0.743}_{(\mathbf{0.65},\mathbf{0.81})}$
NV	$\mathbf{0.740}_{(\mathbf{0.63},\mathbf{0.82})}$	$0.735_{(0.61,0.82)}$	$0.737_{(0.63,0.83)}$
BCC	$\mathbf{0.710}_{(\mathbf{0.54},\mathbf{0.79})}$	$0.694_{(0.47,0.78)}$	$0.670_{(0.50,0.78)}$
AKIEC	$\mathbf{0.748}_{(\mathbf{0.61},\mathbf{0.84})}$	$0.721_{(0.60,0.80)}$	$0.707_{(0.60,0.80)}$
BKL	$\mathbf{0.747}_{(\mathbf{0.58},\mathbf{0.82})}$	$0.724_{(0.61,0.80)}$	$0.732_{(0.60,0.79)}$
DF	$0.381_{(0.32,0.45)}$	$\mathbf{0.736}_{(\mathbf{0.56},\mathbf{0.76})}$	$0.696_{(0.57,0.73)}$
VASC	$0.669_{(0.64,0.73)}$	$\mathbf{0.765}_{(\mathbf{0.52},\mathbf{0.87})}$	$0.692_{(0.50,0.81)}$
OB	$0.566_{(0.31,0.74)}$	$0.559_{(0.08,0.73)}$	$\mathbf{0.634}_{(\mathbf{0.41},\mathbf{0.69})}$

Supplementary Table 2. Sub-type lesion detection from smartphone (wide-field) and dermoscopy images. Performance is intersection over union (IoU) summarized as median (interquartile range).

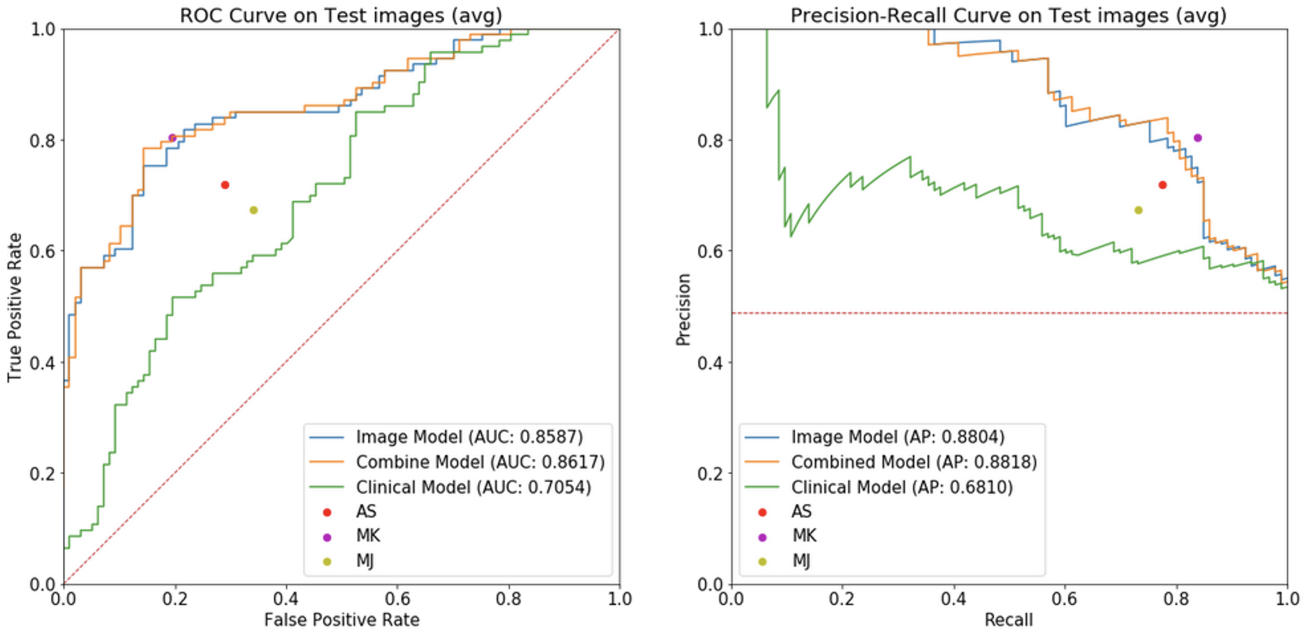

Supplementary Figure 2. Performance metrics of the malignancy prediction models including clinical covariates but without self-report race covariates. ROC and PR curves for three models are presented, namely, combined (clinical + images), image only and clinical covariates only. Also reported are the TPR (sensitivity) and FPR (1-specificity) for three dermatology trained MDs (AS, MK and MJ)
